# Predictive ability of the G8 screening test to determine probable sarcopenia and abnormal comprehensive geriatric assessment in older patients with solid malignancies

**DOI:** 10.1186/s12877-021-02544-9

**Published:** 2021-10-19

**Authors:** Cagatay Cavusoglu, Gozde Tahtaci, Rana Tuna Dogrul, Ibrahim Ileri, Funda Yildirim, Burcu Candemir, Muhammet Cemal Kizilarslanoglu, Aytug Uner, Berna Goker

**Affiliations:** 1 Faculty of Medicine, Department of Internal Medicine, Division of Geriatric Medicine, Gazi University, Ankara, Turkey; 2 Faculty of Medicine, Department of Internal Medicine, Division of Oncology, Gazi University, Ankara, Turkey; 3 Konya Education and Research Hospital, Department of Internal Medicine, Division of Geriatrics and Palliative Care, University of Health Sciences, Konya, Turkey

**Keywords:** G8 screening tool, Probable sarcopenia, Predictive ability, CGA

## Abstract

**Background:**

Pre-treatment evaluation for sarcopenia is recommended in cancer patients. New screening tests that are less time-consuming and can identify patients who will potentially benefit from geriatric assessment are being developed; the G8 geriatric screening test is one such example. We aimed to investigate whether the G8 screening test can detect probable sarcopenia and is valid and reliable compared to a comprehensive geriatric assessment (CGA) in Turkish older adults with solid cancers.

**Methods:**

We included solid cancer patients referred to a single center. Probable sarcopenia and abnormal CGA were defined as low handgrip strength. Cut-offs for handgrip strength in the Turkish population have been previously determined to be 32 kg for males and 22 kg for females and impairment in at least one of the CGA tests, respectively. The CGA tests comprised KATZ Basic Activities of Daily Living Scale Lawton–Brody Instrumental Activities of Daily Living Scale, Mini-Mental-State Examination Scale, Geriatric Depression Scale-15, and Mini-Nutritional Assessment Short Form. Receiver operating characteristic curve analyses evaluated the test’s predictive ability. Intra-rater and inter-rater reliabilities were assessed.

**Results:**

The median age of the 76 patients included was 72 (65–91) years. There was a moderate correlation between handgrip strength and the G8 test total score. The sensitivity and specificity of the G8 test to detect probable sarcopenia alone (cut off score = 12.5) were 50 and 92%, respectively (AUC: 0.747; *p* < 0.001); to determine abnormal CGA plus probable sarcopenia (cut off score = 13) were 93.33 and 86.89%, respectively (AUC: 0.939; *p* < 0.001); and to detect abnormal CGA alone (cut off score = 14) were 79.63 and 95.45%, respectively (AUC: 0.893; *p* < 0.001). The G8 test results agreed with those of CGA (κ = 0.638; p < 0.001). Both inter- and intra-rater assessments of G8 scores revealed a strong agreement (Interclass correlation coefficient = 0.979, *p* < 0.001 and ρ = 0.994, p < 0.001, respectively).

**Conclusions:**

The Turkish version of the G8 test is a good screening tool to detect probable sarcopenia alone and in conjunction with abnormal CGA in older patients with solid malignancies. The G8 screening tool may thus be useful in detecting probable sarcopenia in Turkish older adults with solid cancers.

## Introduction

Oncologists must consider the possibility of adverse events like chemotherapy-induced toxicities, hospitalization, and early death when planning cancer treatment in older adults [1, 2]. Comprehensive Geriatric Assessment (CGA) is the gold standard for determining factors, such as functional, psychosocial, cognitive, vulnerability, and nutritional statuses, in older adults and can be also used in long-term follow-ups [3–5]. The CGA of cancer patients contributes significantly to the individualization of the treatment plan. CGAs are shown to improve the disease-related outcome, increase treatment efficacy, minimize chemotherapy-induced toxicity, and decrease the risk of falls among cancer patients. Moreover, the rates of morbidity, mortality, and unplanned hospitalization are shown to decrease when CGA is implemented in oncological patients [6].

New screening tests that are less time-consuming and can identify patients who will potentially benefit from geriatric assessment are being developed; the G8 geriatric screening test is one such example [7]. The G8 geriatric screening test, developed by Bellera et al. in 2012, assesses the nutritional status, mobility, age, general health status, neuropsychological status, and drug usage in cancer patients [7]. The National Comprehensive Cancer Network guideline for the evaluation of geriatric cancer patients, published in 2020, suggested that geriatric patients should be pre-evaluated with geriatric screening tools such as the G8 geriatric screening tool. In the absence of impairment, measured using these tools, the patient’s treatment should be initiated, whereas CGA should be recommended if the tools indicate an impairment [8]. Although the G8 geriatric screening test is successful in indicating the need for CGA, its ability to screen for sarcopenia has not yet been investigated. In recent years, pre-treatment evaluation for sarcopenia along with CGA has been recommended in cancer patients. An appropriate approach to sarcopenia can improve clinical outcomes in cancer patients [9].

Sarcopenia is the loss of muscle mass, which leads to a reduction in a person’s strength [10]. The prevalence of sarcopenia may vary from 11 to 74% in cancer patients, and these rates are estimated to be much higher in geriatric patients [11]. A meta-analysis showed that sarcopenia causes an increase in treatment-related side effects, postoperative complications, and mortality rates and a decrease in overall survival and disease-free survival in cancer patients [12]. In cases where gold standard diagnostic methods are unavailable for sarcopenia, handgrip strength may be used for probable sarcopenia [13]. The Consortium of Sarcopenia Definition and Outcomes stated that reduced handgrip strength is related to dependence, falls, and worse outcomes in daily activities [10]. Kilgour et al. showed that handgrip strength was independently related with quality of life, performance status, clinical parameters, and mortality in cancer patients [14]. Chaucan et al. suggested that neck cancer patients should be screened, and those with low handgrip strength on follow-up should be given appropriate nutritional exercises and treatment [15]. Although sarcopenia testing is recommended in all oncological outpatients [16], staff, well-trained in geriatrics, are needed for this assessment process [17]. Moreover, no studies have yet investigated the relationship with sarcopenia and G8 screening test in solid cancer patients.

In this study, we primarily aimed to evaluate whether the G8 screening test could detect probable sarcopenia. Secondarily, we examined the validity and reliability of the G8 screening test in Turkish solid cancer patients by comparing it to the gold standard (CGA).

## Materials and methods

### Study sample and design

This study was conducted at the outpatient clinics of oncology and geriatrics at the Gazi University Hospital. We enrolled 148 consecutive patients with solid cancers, who were followed up at the outpatient clinic of oncology and were referred to the geriatric outpatient clinic. Of these, 45 patients, who had previously received chemotherapy or radiotherapy, were excluded because evaluating recipients and non-recipients of cancer treatment concurrently may create a bias. Additionally, CGA should ideally be performed before or during treatment planning [16, 18]. Therefore, we planned to perform the G8 geriatric screening test before the treatment to identify patients who will benefit from CGA. Twelve patients were excluded from the study owing to Alzheimer’s disease. Seven patients were excluded owing to visual and auditory problems. The study was conducted with the remaining 76 patients. The test was performed by a geriatrician through a face-to-face interview in a separate room in the geriatric outpatient clinic. The sociodemographic characteristics, educational status, concomitant chronic diseases, medication, urinary incontinence, history of fall in the last year, and the number of prescribed drugs were recorded. First, the G8 geriatric screening test was performed, followed by a CGA. The CGA comprised KATZ Basic Activities of Daily Living Scale (BADL), Lawton–Brody Instrumental Activities of Daily Living Scale (IADL), Mini-Mental-State Examination Scale (MMSE), Geriatric Depression Scale-15 (GDS-15), and Mini-Nutritional Assessment Short Form (MNA-SF). We determined that a patient had an abnormal CGA, if they had an abnormal score in at least one of the CGA tests (BADL score ≤ 5; IADL score ≤ 7, MNT-SF score ≤ 11, MMSE score ≤ 23, and GDS-15 score ≥ 6). Thereafter, the patients’ handgrip strength was measured.

### Ethical consideration

The study was approved by the Gazi University Ethics Committee for Clinical Research. Written informed consent was obtained from all participants (reference no: E.133043). Permission to use an adaptation of G8 screening tool was obtained from the investigators (C. Bellera).

### Comprehensive geriatric assessment parameters

#### KATZ basic activities of daily living scale

The BADL scale has six dimensions: bathing, dressing, toileting, transport at home, continence, and feeding and assesses whether the individual is independent during his/her daily activities [19]. A score of 6 in its 6-point assessment scale indicates that the patient is fully independent during the basic daily activities [19]. A score of ≤5 indicates that independence during daily activities is impaired. Therefore, we considered a BADL score ≤ 5 as an abnormal CGA. The validity and reliability of this tool for the Turkish language was confirmed previously [20].

#### Lawton–Brody instrumental activities of daily living scale

The Lawton- Brody IADL Scale has eight dimensions: shopping, telephone, laundry, housekeeping, food preparation, transport, medication, and finances. The score ranges between 0 and 8, with 8 points indicating full independence in daily activities [21]. Isik et al. validated the Turkish version Lawton-Brody IADL [22]. We accepted a score ≤ 7 as an abnormal CGA.

#### Mini-mental state examination

This test assessed cognitive abilities with scores ranging between 0 and 30; a score ≥ 24 indicated normal cognitive function [23]. The validation of MMSE for the Turkish language has been previously conducted [24]. An MMSE score < 24 was considered an abnormal CGA in our study.

#### Mini nutritional assessment short form

The MNA-SF evaluates nutritional status and malnutrition risk, and has a scoring range between 0 and 14 points. A score > 11 indicates normal nutritional status [25]. The validity and reliability of both short and long forms for the Turkish language have already been conducted [26]. We considered a score ≤ 11 as an abnormal CGA.

#### Geriatric depression Scale-15

This scale assesses depressive symptoms in geriatric patients. The GDS-15 consists of 15 questions (yes/no) that assess the energy of the patients during the day, happiness in life, hopelessness, loneliness, anxiety and worthlessness. The total score of this self-report scale may vary between 0 and 15. A score ≥ 5 indicates depression risk [27]. The validity and reliability of GDS-15 for the Turkish language have been evaluated previously [28]. We considered a score ≥ 5 as an abnormal CGA.

### Frailty assessment

The Clinical Frailty Scale (CFS) assessed patients’ frailty, and its Turkish validity and reliability study was conducted by Ozsurekci et al. The CFS score ranges from 1 to 9, and the scoring is based on the physician’s clinical opinion; while 1 point means a very fit person, 9 points mean terminally ill. The higher the score, the higher the level of vulnerability [29, 30].

### Sarcopenia assessment

When muscle strength is evaluated primarily using handgrip, patients with low handgrip strength are defined to have probable sarcopenia [13]. A digital handheld dynamometer (T.K.K.5401; Takei Scientific Instruments, Tokyo, JAPAN) was used to evaluate muscle mass. While the patients sat on a chair with their arms parallel to the floor, they squeezed the dynamometer thrice with the dominant hand and the maximum value was obtained. The cut-offs for handgrip strength in the Turkish population were previously determined to be 32 kg for males and 22 kg for females [31]. We grouped patients as non-sarcopenic or sarcopenic according to whether their maximum value exceeded the respective cut-offs.

### G8 geriatric screening test

The G8 screening test, an easy-to-use tool to screen the need for CGA, consists of eight questions and a scoring range between 0 and 17 points. A score ≤ 14 suggests abnormal results and indicates that the patient may benefit from a CGA. Accordingly, we accepted a score of 14 as the cut-off value [7, 32].

#### Reliability

To assess intra-rater reliability, the scale was reapplied to 14 individuals 7–10 days after the first application. Fourteen patients were selected for the inter-rater reliability, and the G8 test was performed by another geriatrist in a separate room.

#### Translation into Turkish

The translation of the G8 geriatric screening test was performed by two independent translators using the methodology of forward and backward translation. The final translation was reviewed and compared by clinicians to assume both item and semantic equivalence. The translation was tested on a small group of patients to assess their conceptual understanding of it.

## Statistical analysis

Chi-square or Fisher’s exact tests were used to compare categorical variables that are presented as numbers and percentages (n, %). Distribution of numerical parameters were evaluated using histogram, coefficients of variation, and Kolmogorov–Smirnov tests. Normally and non-normally distributed numerical parameters are presented as mean ± standard deviation (SD) and median (minimum-maximum values), respectively. Normally and non-normally distributed numerical parameters were compared between two independent groups using the Student’s t-test and Mann–Whitney U test, respectively. Because of the non-normal distribution of data, the correlation between the total G8 score and CGA parameters was analyzed with the Spearman’s test. The correlation analysis between two categorical variables was performed using the k method. Receiver operating characteristic (ROC) evaluation was made according to three different levels to evaluate the performance of the G8 geriatric screening test at diagnosis: 1.) abnormal CGA was examined with G8, 2.) probable sarcopenia was examined with G8, and 3.) patients were examined with CGA and for probable sarcopenia. If the area under the curve (AUC) was close to 1, the ability to predict probable sarcopenia was considered to have excellent diagnostic accuracy, and the sensitivity and specificity values were determined. Following the analysis, the Youden index was calculated to determine the optimal cut-off value. Positive and negative predictive values were calculated. For the evaluation of the test-retest and inter-clinician reliability, 14 separate patients were selected for each of these items, and the performed G8 geriatric screening test was assessed with the k analysis. Spearman’s analysis was used to evaluate the correlation between the total scores.

## Results

The median age of the study population was 72 years (65–91 years). Of the total patients, 43% were female, 16% were literate, and 30% were educated for ≤5 years. The general characteristics of the patients are listed in Table 1.

**Table 1:** General characteristics of the patients

	Male (*n* = 43)	Female (*n* = 33)
Age, year, median (min-max)	72 (62–91)	71 (65–90)
Number of drugs, number (min-max)	3 (0–10)	3 (0–11)
Number of chronic diseases, number, median (min-max)	2 (1–6)	3 (1–6)
BMI, kg/m^2^, mean ± SD	27 ± 4.4	29 ± 5.2
BADL score, median (min-max)	6 (2–6)	6 (4–6)
IADL score, median (min-max)	8 (0–8)	8 (5–8)
MMSE score, median (min-max)	29 (15–30)	28 (15–30)
MNA-SF score, median (min-max)	13 (5–14)	12 (6–14)
GDS-15, median (min-max)	2 (0–14)	1 (0–12)
CFS, median (min-max)	2 (1–8)	3 (1–8)
Handgrip strength, median (min-max)	30 (11.7–45.4)	17.8 (9.8–28)
Probable sarcopenic^a^, number, percentage	15 (%53.6)	13 (%46.4)
G8 total score, median (min-max)	14 (6–17)	14 (8–17)

*BMI* Body Mass Index, *BADL* Basic Activities of Daily Living, *IADL* Instrumental Activities of Daily Living, *MMSE* Mini-Mental State Examination, *MNA-SF* Mini Nutritional Assessment Short Form, *CFS* Clinical Frailty Scale

^a^: Gender based cut-offs were used to detect probable sarcopenia

Genitourinary, breast, colon, and lung cancers were seen in 39.5, 30.3, 21.1, and 9.2% of the patients, respectively. In 71% of patients, at least one of the CGA parameters was abnormal, and 36.8% of the patients had probable sarcopenia. The total G8 score was ≤14 in 43.4% of patients. All participants had at least one chronic co-morbidity. The median value of the prescribed medications was 3 (range, 0–11). The correlation between handgrip strength and G8 test score was moderate. There was a strong negative correlation between the patients’ G8 total score and CFS scores (Table 2).

**Table 2:** Correlation between the G8 test score, Comprehensive Geriatric Assessment (CGA) tests, handgrip strength and Clinical Frailty Scale (CFS)

	G8 Spearman’s Rho Coefficient	*P*-value
BADL	0.241	p < 0.001
IADL	0.409	p < 0.001
MMSE	0.411	p < 0.001
MNA-SF	0.657	p < 0.001
GDS-15	−0.391	p < 0.001
Number of falls last year	−0,550	p < 0.001
Handgrip strength	0.527	p < 0.001
CFS	−0.771	p < 0.001

*BADL* Basic Activities of Daily Living, *IADL* Instrumental Activities of Daily Living, *MMSE* Mini-Mental State Examinationt, *GDS* Geriatric Depression Scale, *MNA-SF* Mini Nutritional Assessment Short Form, *GDS* Geriatric Depression Scale, *CFS* Clinical Frailty Scale

Considering the assessment of validity, we found moderate-to-high conformity of the normal and abnormal categorization by the G8 geriatric screening test (≤ 14 abnormal) to that by the CGA (κ = 0.638; *p* < 0.001). Inter- and intra-observer agreements in the reliability assessment were significantly high (κ = 0.837; κ = 0.857; *p* < 0.001, respectively). Both inter- and intra-clinician assessments of G8 scores showed a statistically significant correlation (r = 0.965 and r = 0.982, respectively; p < 0.001).

**Fig. 1:**
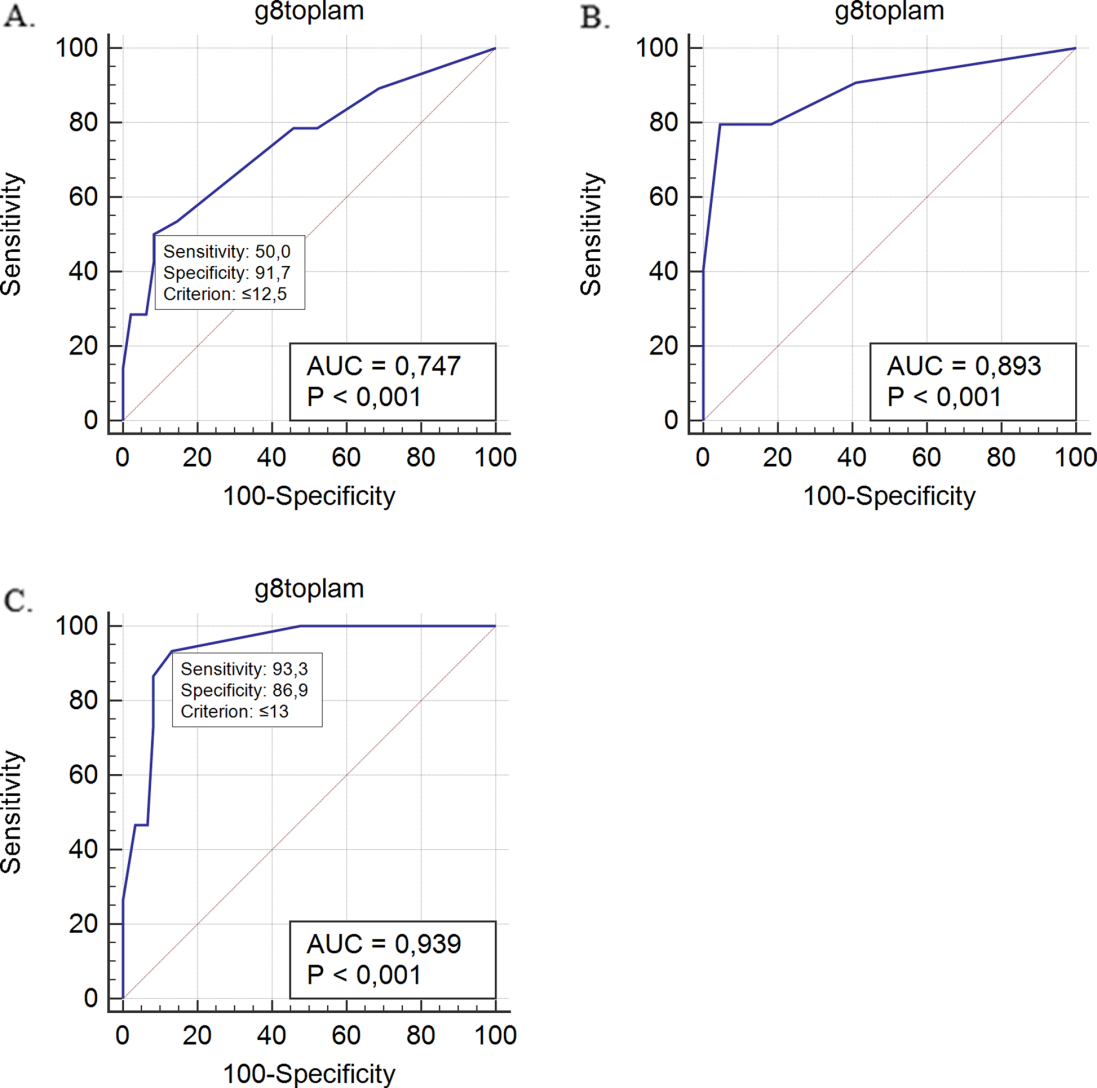
ROC analyses of the G8 screening test. **A**. Probable sarcopenia; **B**. Abnormal Comprehensive Geriatric Assessment; **C**. Abnormal Comprehensive Geriatric Assessment and probable sarcopenia. AUC, Area under the curve; ROC, receiver operating characteristic curve

Correlations between the G8 test score and individual components of the CGA revealed a weak correlation with BADL; moderate correlations with IADL and MMSE; a moderate-to-strong correlation with MNT-SF; a weak-to-moderate negative correlation with GDS-15; and a negative moderate correlation with the number of falls in the last year (Table 2). While the G8 screening score was 12 (range, 6–17) in patients who fell within the previous year, it was 15 (range, 7–17) in those who did not (*p* < 0.001). The results of the three different ROC analyses performed for the G8 geriatric screening test are shown in Fig. 1.

The sensitivity, specificity, and positive and negative predictive values of the G8 test for different cut-off values are given in Table 3.

**Table 3:** The G8 screening test: sensitivity, specificity, and cut-off values

Criterion	Sensitivity	95% CI	Specificity	95% CI	PPV	95% CI	NPV	95% CI
The G8 sensitivity and specificity for different cutoff values about probable sarcopenia								
≤11	28.57	13.2–48.7	93.75	82.8–98.7	72.7	43.5–90.2	69.2	63.8–74.2
≤12	42.86	24.5–62.8	91.67	80.0–97.7	75.0	51.7–89.4	73.3	66.4–79.3
≤12.5^a^	50.00	30.6–69.4	91.67	80.0–97.7	77.8	56.1–90.6	75.9	68.2–82.1
≤13	53.57	33.9–72.5	85.42	72.2–93.9	68.2	49.9–82.2	75.9	67.6–82.7
≤14	78.57	59.0–91.7	54.17	39.2–68.6	50.0	41.0–59.0	81.2	67.1–90.2
The G8 sensitivity and specificity for different cutoff values about abnormal CGA								
≤12.5	33.33	21.1–47.5	100.00	84.6–100.0	100.0		37.9	33.6–42.5
≤13	40.74	27.6–55.0	100.00	84.6–100.0	100.0		40.7	35.5–46.2
≤14^b^	79.63	66.5–89.4	95.45	77.2–99.9	97.7	86.3–99.7	65.6	52.8–76.5
≤15	79.63	66.5–89.4	81.82	59.7–94.8	91.5	81.4–96.3	62.1	48.2–74.2
≤16	90.74	79.7–96.9	59.09	36.4–79.3	84.5	76.6–90.1	72.2	51.3–86.5
The G8 sensitivity and specificity for different cutoff values about abnormal CGA and probable sarcopenia								
≤12	73.33	44.9–92.2	91.80	81.9–97.3	68.7	47.4–84.3	93.3	85.8–97.0
≤12.5	86.67	59.5–98.3	91.80	81.9–97.3	72.2	52.3–86.0	96.6	88.5–99.0
≤13^c^	93.33	68.1–99.8	86.89	75.8–94.2	63.6	47.5–77.2	98.1	88.8–99.7
≤14	100.00	78.2–100.0	52.46	39.3–65.4	34.1	28.4–40.2	100.0	
≤15	100.00	78.2–100.0	47.54	34.6–60.7	31.9	27.0–37.3	100.0	

*CI* Confidence interval, *PPV* Positive predictive value, *NPV* Negative predictive value

^a,b,c^ Cutoff value that corresponded to the highest Youden index in the receiver operating characteristic curve analysis

When three components of the G8 geriatric screening test (weight loss, BMI [Body Mass Index], and mobility) were chosen to detect the sensitivity/specificity for probable sarcopenia, the *p* value was not statistically significant.

## Discussion

Our findings show that the G8 test score has high power in detecting CGA impairment and probable sarcopenia. Moreover, this tool is successful in identifying probable sarcopenia exclusively. To the best of our knowledge, this study is the first to evaluate probable sarcopenia using the G8 screening exclusively and in conjunction with CGA.

Preparing a treatment plan for cancer patients requires careful consideration of several factors, such as the patients’ physical status, performance, cognitive function, nutritional status, frailty, sarcopenia, and risk of chemotherapy-induced toxicities. All these factors directly or indirectly affect the mortality and morbidity of the patient [16, 18, 33]. CGA and sarcopenia detection are helpful tools for physicians to identify patients who require additional care. However, CGA is a time-consuming approach and requires specially trained staff to conduct it [17].

Ryan et al. showed that sarcopenia is associated with reduced tolerance to chemotherapy, increased postoperative complications, impaired quality of life, and shorter lifespan in cancer patients [34]. Of the different methods used to detect sarcopenia, tests, such as Dual-Energy X-ray Absorptiometry (DEXA), Computed Tomography (CT), Magnetic Resonance Imaging (MRI) that show muscle mass, are considered the gold standard. In the absence of these diagnostic tools, handgrip strength can be used to detect probable sarcopenia [31]. Kilgour et al. showed that handgrip strength was independently associated with survival, functionality, and quality of life [14]. Our results corroborate these findings as we detected that patients with muscle strength also had a low G8 score. Moreover, we showed that as the G8 score decreased, the number of falls in a year increased, whereas the BADL, IADL, and cognition scores reduced. Therefore, we infer that the G8 test score is associated with patients’ quality of life and functional independence. G8’s items about mobility, BMI, and weight loss were insufficient to detect any cut off scores for probable sarcopenia. But our first priority was to evaluate the overall success of the G8 test in predicting sarcopenia. Considering the screening power of the G8 score for probable sarcopenia when the cut-off value was taken as 12.5, the sensitivity was low (50%) but the specificity was high (92%). The G8 test’s sensitivity in diagnosing probable sarcopenia was not at the expected level. This could lead to a low number of false positives.

We found a strong correlation between the G8 test and the CGA (κ 0.638; *p* < 0.001). To consider the G8 test as reliable, it should provide consistent results when performed by different persons and by the same person at different times. Both the inter-clinician assessments and the assessments repeated by the same clinician at different times provided consistent results and were strongly correlated with the CGA. While investigating the validity and reliability of the G8 geriatric screening test in cancer patients, the calculated cut-off values should be compared with those referred in the original study. Although the cut-off value in our study (≤ 14) according to the ROC analysis was similar to that in the original study [7], the specificity (95%) and sensitivity (79%) rates found in our study differed slightly from those seen in previous studies. In the study conducted by Bellera et al. [7] with the same cut-off value [14], the sensitivity and specificity rates were 85 and 65%, respectively. Although the sensitivity rates were comparable, the specificity rate was higher in our study. During the calculation of abnormal CGA, Bellera et al. [7] included the deterioration in the Cumulative Illness Rating Scale-Geriatric (CIRS-G) and Timed Up and Go (TUG) tests into the calculation in addition to the test used by us. The differences between the sensitivity and specificity rates in these two studies may depend on this factor. The cut-off value was 12.5 in the study conducted by Baitar et al., and the components of GCA, as well as the definition of abnormal CGA used by them, differed from ours; they used the GDS-30, social support index, and timed-get up and go tests for CGA [35].

Since both are frailty indices, a strong correlation between CFS and G8 was expected. However, one of the disadvantages of CFS is that it is subjective and varies from person to person. On the other hand, if more research supports the G8’s relationship with frailty and probable sarcopenia, this could be one of the G8’s benefits [36].

The fact that the G8 test detects those with both probable sarcopenia and impaired CGA (AUC = 0.939) indicates that the detection rate of the test is outstanding. Although its sensitivity and specificity were found to be adequate (93, 86%, respectively), its negative predictive value of 98.1%, which detects the distinction of healthy individuals, could qualify the G8 screening tool as a potentially ideal screening test [37].

In our study, we also investigated the correlation of the G8 test scores with the sub-groups of the CGA. Individuals’ physical and functional limitations play an essential role in determining chemotherapy. Increased dependence was found to be associated with a shorter lifespan [38–40]. The cognitive level of the cancer patient may play an important role in deciding the treatment plan, evaluating chemotherapy-induced toxicity, and determining the most appropriate approach together with the patient [41]. In addition, the survival and mortality rates are significantly affected by impaired cognitive function and low MMSE scores [42, 43]. Therefore, cognitive function should definitely be assessed in cancer patients [41]. Cancer leads to many adverse changes in the patient’s life. Limitations in social life, job loss, sexual dysfunction, change in the meaning of life, and financial problems may arise and negatively impact the psychological status of the patient [44]. The concomitant depression in older adults with cancer worsens the loss of appetite, weight loss, and malnutrition associated with malignancy. Mood disorders decrease the functionality and quality of life and more importantly, compliance with the cancer treatment [45]. In older adults, depression is also associated with increased mortality [44, 46–48]. Weinberger et al. emphasize that depression was very common among cancer patients and led to negative outcomes [49]. The American Society of Clinical Oncology recommended a routine and regular examination of the mood status in cancer patients [44]. The correlation between G8 and CGA sub-parameters (ADL, IADL, MMSE, MNA -SF, GDS − 15) makes G8 clinically more critical. Hamaker et al. showed that G8 is essential in determining survival in patients with hematologic cancer [50]. Takashi et al. showed that it is associated with poor prognosis and that adding G8 and Eastern Cooperative Oncology Group Performance Status (ECOG-PS) be more beneficial [51].

Another important aspect of this study is its novelty in evaluating the usability of the G8 screening test in detecting probable sarcopenia. Although the specificity of the G8 screening test in detecting probable sarcopenia is high, the high sensitivity and specificity of this test in evaluating CGA and sarcopenia together is particularly notable. The study’s inadequacies comprise the small sample size and evaluation of only probable sarcopenia. Besides, the concomitant co-morbidities could be included in CGA with the scores calculated with the help of one of the co-morbidity indexes.

Our secondary aim was to determine the validity and reliability of the G8 test. Our results show that compared with the CGA, the Turkish version of the G8 test is a valid and reliable screening tool for solid cancer patients. The inclusion of different types of solid cancers in this study improves the generalizability of our findings. Further, the implementation of the CGA by a geriatrist instead of specialists from other fields increases the reliability of the tests used. The correlations of the G8 test with both the CGA and its individual components determined in our study indicate that this test can be safely used in cancer patients in our country.

The study’s main limitations were its limited number of patients, cross sectional, descriptive nature, and single-center design. Prospective markers such as chemotherapy toxicity, hospitalization, or mortality could not be assessed because the patients were not followed up after the initial evaluation. The inability to measure muscle mass death or psoas area determined by imaging techniques such as CT, DEXA, Bioelectrical Impedance Analysis (BIA) or MRI is one of the limitations of our study. The use of performance tests to assess the severity of sarcopenia is recommended. Since tests like the TUG and 6 m walk test were not used in our study, the severity of sarcopenia could not be assessed. Furthermore, as proposed by the European and/or Asian consensus, lower limb muscle performance and Strength, Assistance with walking, Rising from a chair, Climbing stairs, and Falls (SARC-F)/calf-circumference could be used to evaluate lower extremity muscles.

## Conclusion

The G8 test may not be the best choice to evaluate probable sarcopenia alone because of low sensitivity. Instead, The G8 test is more successful in detecting CGA and probable sarcopenia together. Also, the G8 test is closely correlated with abnormal CGA. Future studies should investigate the relationship between sarcopenia and the G8 test comprehensively. The G8 test may also be compared with different vulnerability scales and sarcopenia screening tests, and prospective studies could help investigate its long-term predictive value.

## Data Availability

Data is kept confidential. The datasets generated and/or analyzed during the current study are available from the corresponding author on reasonable request.
